# Inhibition of mTORC1 inhibits lytic replication of Epstein-Barr virus in a cell-type specific manner

**DOI:** 10.1186/1743-422X-11-110

**Published:** 2014-06-11

**Authors:** Amy L Adamson, Brandi T Le, Brian D Siedenburg

**Affiliations:** 1 Department of Biology, University of North Carolina at Greensboro, Greensboro, North Carolina 27402, USA

**Keywords:** Epstein-Barr virus, BZLF1, BRLF1, Lytic replication, mTOR, Rapamycin

## Abstract

**Background:**

Epstein-Barr virus is a human herpesvirus that infects a majority of the human population. Primary infection of Epstein-Barr virus (EBV) causes the syndrome infectious mononucleosis. This virus is also associated with several cancers, including Burkitt’s lymphoma, post-transplant lymphoproliferative disorder and nasopharyngeal carcinoma. As all herpesvirus family members, EBV initially replicates lytically to produce abundant virus particles, then enters a latent state to remain within the host indefinitely.

**Methods:**

Through a genetic screen in *Drosophila*, we determined that reduction of *Drosophila Tor* activity altered EBV immediate-early protein function. To further investigate this finding, we inhibited mTOR in EBV-positive cells and investigated subsequent changes to lytic replication via Western blotting, flow cytometry, and quantitative PCR. The student T-test was used to evaluate significance.

**Results:**

mTOR, the human homolog of *Drosophila* Tor, is an important protein at the center of a major signaling pathway that controls many aspects of cell biology. As the EBV immediate-early genes are responsible for EBV lytic replication, we examined the effect of inhibition of mTORC1 on EBV lytic replication in human EBV-positive cell lines. We determined that treatment of cells with rapamycin, which is an inhibitor of mTORC1 activity, led to a reduction in the ability of B cell lines to undergo lytic replication. In contrast, EBV-positive epithelial cell lines underwent higher levels of lytic replication when treated with rapamycin.

**Conclusions:**

Overall, the responses of EBV-positive cell lines vary when treated with mTOR inhibitors, and this may be important when considering such inhibitors as anti-cancer therapeutic agents.

## Background

Epstein-Barr virus (EBV) is a human herpesvirus which has infected a large majority of the world’s human population. This virus infects epithelial cells of the nasopharynx, where it replicates in a lytic or productive manner, as well as B lymphocytes, where the virus enters a latent state [1]. EBV is associated with a plethora of diseases, including infectious mononucleosis, lymphomas (Burkitt’s lymphoma, Hodgkins lymphoma, post-transplant lymphoproliferative disorder), epithelial-based cancers (including nasopharyngeal carcinoma), multiple sclerosis, and the rare but deadly X-linked lymphoproliferative syndrome [2]. The prevalence of this virus, along with its potential for serious disease, necessitate the study of means to inhibit lytic replication, as well as treatments to kill EBV-positive cancer cells.

EBV lytic replication commences upon infection of new cells, or upon reactivation of the latent virus within cells. During lytic replication the first wave of EBV protein activity includes the immediate-early BZLF1 (Z) and BRLF1 (R) proteins. The Z and R proteins are both absolutely necessary for lytic replication to proceed. These are transcription factors that activate transcription from the promoters of the next wave of EBV genes, the early genes. The early genes encode proteins that act to replicate the viral genome. Lastly, the late genes are expressed to provide the virion structural elements [1].

The Z and R proteins act not only as transcriptional activators, but also physically interact with several cellular proteins including CREB-binding protein (CBP), in order to promote viral replication in lieu of cellular activities [3,4]. Z and R have also been found to activate MAPK pathways, including the p38, JNK, and ERK pathways [5-7]. Activation of these pathways has been found to be essential for EBV lytic replication. In addition, both Z and R are SUMO-1 modified, which negatively affects Z transcriptional activity, while enhancing R transcriptional activity [8-11]. A potential means of inhibiting viral replication in cells would be to suppress the activity of Z and/or R. Hindering key viral protein/cellular protein interactions may lead to such suppression of Z and R activities, and therefore inhibit EBV lytic replication.

mTOR is a kinase at the heart of a major signaling pathway. mTOR can be found in two different protein complexes, mTORC1 and mTORC2 [12,13]. Extracellular signals such as various nutrient levels and growth factors impinge on the mTORC1 pathway to control a variety of processes including protein translation, autophagy, cell growth, and mitochondrial metabolism [12-14]. Pathways that activate mTORC1 include the MAPK ERK and Akt signaling pathways, both of which can be activated by phosphatidylinositol 3 kinase (PI3 kinase) [15]. As part of the mTORC1 complex, mTOR promotes the phosphorylation of downstream targets including p70S6K and 4EBP1. These events promote ribosome biogenesis and cap-dependent translation, respectively [13,15,16].

Rapamycin is a specific inhibitor of mTOR activity within mTORC1. Rapamycin, also an immunosuppressant, complexes with the protein FKBP-12; this complex then binds to mTOR and inhibits its kinase activity [16]. As inhibition of mTOR subsequently inhibits protein translation and cell growth, rapamycin is an excellent candidate for anti-tumor treatment. In fact rapamycin (or similar mTOR inhibitors) has gained interest for the treatment of cancers, including EBV-associated post-transplant lymphoproliferative disease [17-19].

Previous studies have shown that inhibition of mTOR by treatment with rapamycin is effective in inhibiting Kaposi’s sarcoma herpesvirus (KSHV) lytic replication [20]. The inhibition of lytic replication appears to be due to the inhibition of translation of the immediate-early protein, RTA [20]. Another mTOR inhibitor, Torin1, was found to inhibit viral replication for the herpesvirus members cytomegalovirus, herpes simplex virus 1, and murine gammaherpesvirus 68 [21]. These three viruses were much less sensitive to rapamycin treatment [21]. In the case of human cytomegalovirus, the inhibition of mTOR did not greatly affect the immediate-early proteins, as for KSHV, but appeared to inhibit downstream replication events [21].

In a *Drosophila* model system, we identified *Tor* as a modifier of Z and R activities. Translating this finding to the context of lytically-replicating EBV, we found that mTORC1 inhibition via rapamycin treatment yielded different effects in B cell versus epithelial cell lines. While rapamycin treatment of EBV-positive B cells inhibited lytic replication, rapamycin treatment increased lytic replication in the EBV-positive epithelial cell lines tested, suggesting that the effects of mTOR inhibition differ greatly, in respect to lytic replication, between different cell types. These effects upon EBV lytic replication appear to be, at least in part, due to differential influences upon Z and R gene expression.

## Results

### Loss of *Tor* modified *GMR-Z* and *GMR-R* phenotypes in *Drosophila*

**Figure 1:**
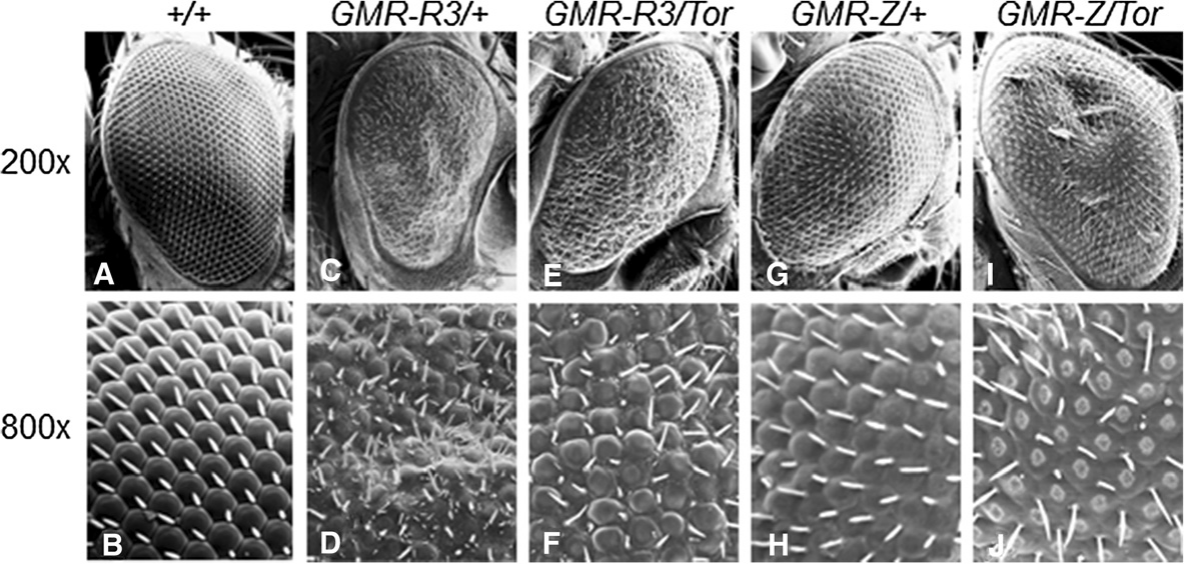
**Loss of *Tor *alters Z and R activity in a *Drosophila *model system. A-B**. Wild-type eye. **C-D**. *GMR-R3* heterozygote. Note the rough eye phenotype and extra small bristles in **D**. **E-F**. *GMR-R3/Tor* transheterozygote. Note the more wild-type structure and reduction of extra small bristles. **G-H**. *GMR-Z* heterozygote. **I-J**. *GMR-Z/Tor* transheterozygote. Note the flattening of the ommatidia in **J**.

We have previously expressed the Z and R genes in *Drosophila* eye tissue, which yielded significant mutant eye phenotypes (Figure 1C, D, G, H) [22,23]. Such phenotypes allowed us to perform genetic screens to identify host cellular modifiers of Z or R activity. One such screen involved crossing our Z and R expressing flies to *Drosophila* tumor suppressor mutants [23]. An interesting finding was that when R-expressing flies (*GMR-R3*) were crossed to *Tor* (the fly homolog of mTOR) mutant fly lines (*Tor*^*DeltaP*^ and *Tor*^*k17004*^, which both decrease Tor levels [24,25]; the *Tor*^*DeltaP*^/+and *Tor*^*k17004*^/+flies alone have wild-type-appearing eyes) the progeny had more wild-type eyes, suggesting that reduced Tor levels suppressed R activity in eye tissue (Figure 1E, F). Conversely, when Z-expressing flies (*GMR-Z*) were crossed to the same *Tor* mutant fly lines, their progeny had a much more severe mutant phenotype, suggesting that the reduction of Tor actually increased Z activity (Figure 1I, J). Comparing the *Tor*-modified *GMR-R3* and *GMR-Z* phenotypes (Figure 1F and J), it appears that the decrease of Tor activity impacted the *GMR-Z* phenotype more so than the *GMR-R3* phenotype. The *GMR-Z/Tor* phenotype is much more severe than *GMR-Z/+*; the *GMR-R3/Tor* phenotype is moderately improved in relation to the *GMR-R3/+p*henotype. This suggests that the loss of *Tor* may impact Z activity more so than R activity.

### Inhibition of mTOR via rapamycin decreases EBV lytic replication in B cell lines, but not in epithelial cell lines

**Figure 2:**
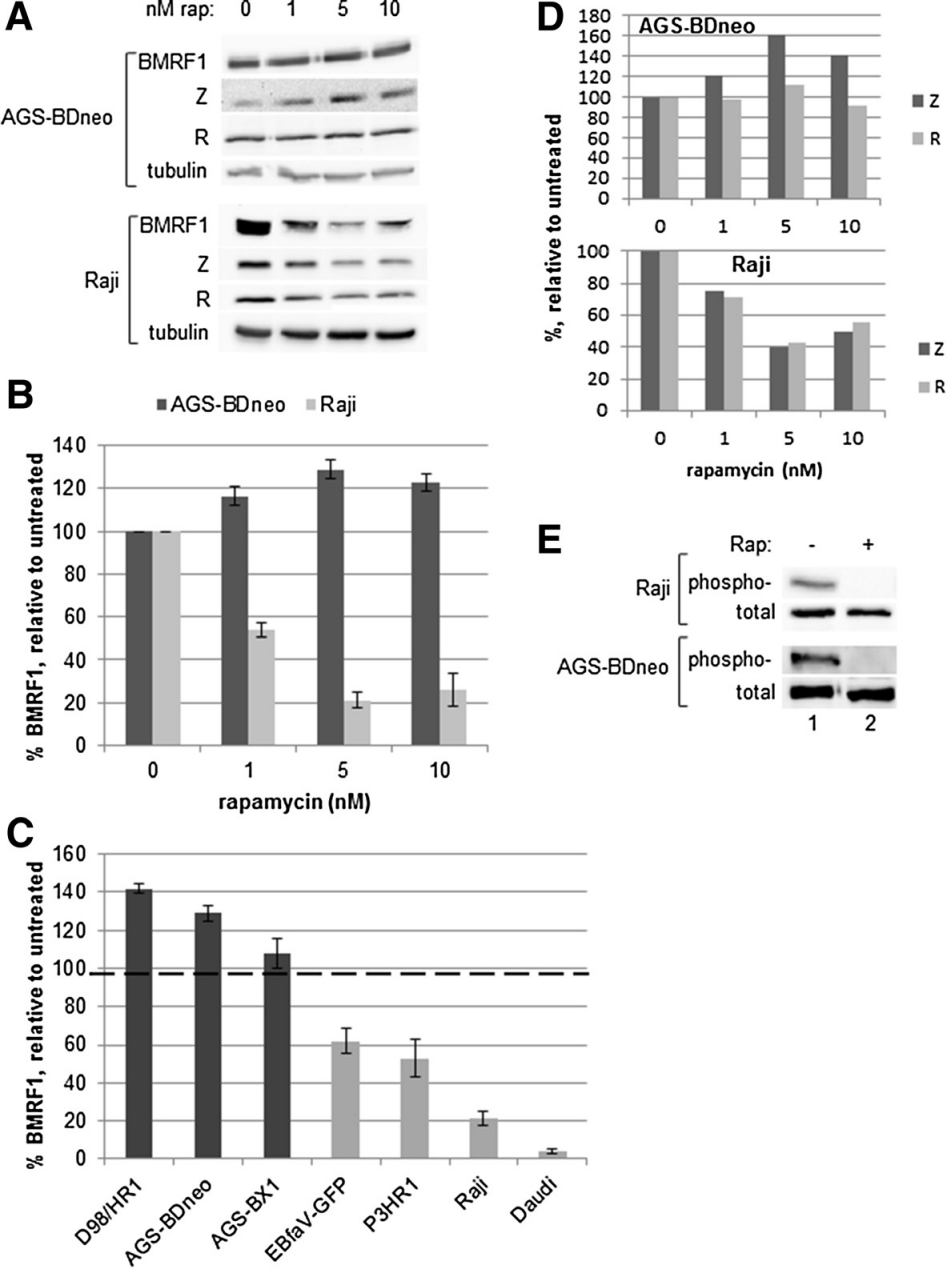
**Inhibition of mTORC1 alters early lytic replication in EBV-positive cells. A**. AGS-BDneo or Raji cells were treated with 0, 1, 5, or 10 nM rapamycin 24 hr prior to induction of lytic replication. Western blot analysis was performed with anti-BMRF1, anti-Z, anti-R, and anti-tubulin antibodies. Representative blots are shown. **B**. The BMRF1 levels from Western blots (in triplicate) were quantified and standardized to tubulin levels. The resulting BMRF1 protein levels are presented, relative to induced cells (with no rapamycin treatment). **C**. BMRF1 levels of a panel of EBV-positive cells treated with 5 nM rapamycin, induced, and analyzed as in parts **A** and **B** above. Dark bars are epithelial cell lines, light bars are B cell lines. **D**. Z and R levels from the Western blots in part **A** were quantified and standardized to tubulin levels. The resulting Z and R protein levels are presented, relative to induced cells (with no rapamycin treatment). **E**. Inhibition of mTOR with 5 nM rapamycin inhibits mTOR activity. Raji and AGS-BDneo cells were treated with 0 or 5 nM rapamycin for 2 days, then subjected to Western blot analysis with anti-phospho-p70S6K and total p70S6K antibodies.

**Figure 3:**
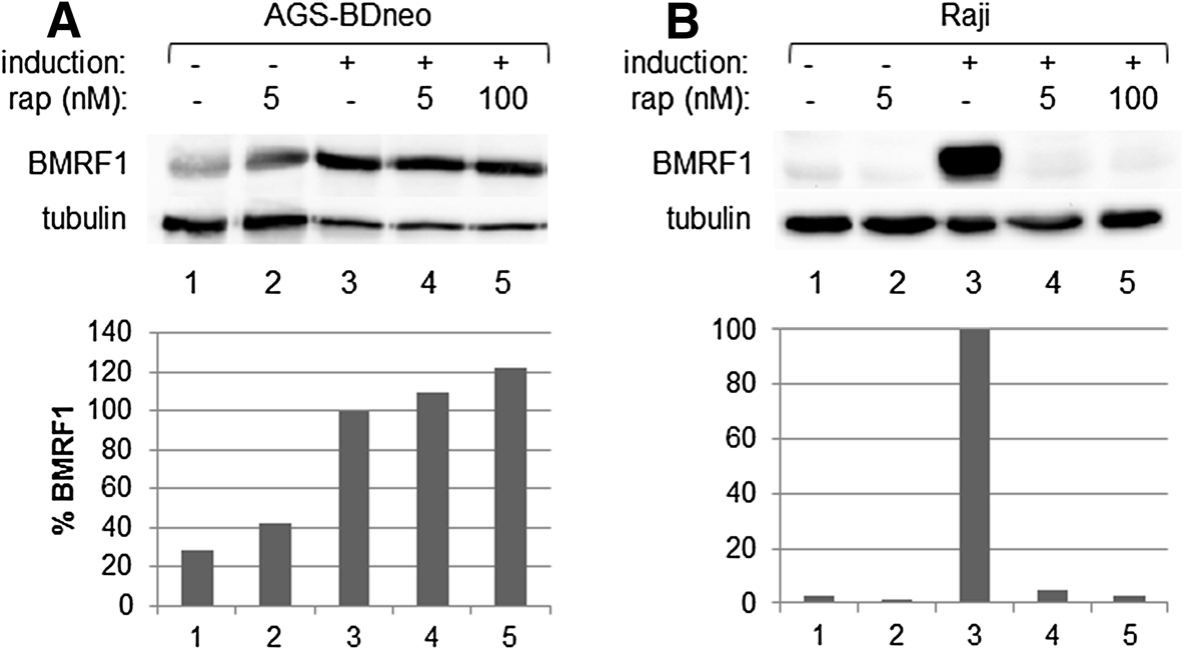
**Higher doses of rapamycin are not effective in inhibiting EBV lytic replication in AGS-BDneo cells. A**, AGS-BDneo cells, **B**, Raji cells. Cells were treated with rapamycin as indicated for 24 hr prior to the induction of lytic replication. Western blot analysis was performed with anti-BMRF1 and anti-tubulin antibodies. The BMRF1 levels were quantified and standardized to tubulin levels. The resulting BMRF1 protein levels are presented, relative to induced cells (with no rapamycin treatment).

As loss of *Tor* affected Z and R activity in *Drosophila* eye cells, we hypothesized that a reduction of mTOR activity in human cells would affect Z and/or R activity and thus alter EBV lytic replication within EBV-positive cells. To this end, we treated the latently-infected, EBV-positive epithelial cell line AGS-BDneo with 0, 1, 5, or 10 nM rapamycin for 24 hr prior to the induction lytic replication. We performed Western blot analyses to examine levels of the early protein BMRF1, an indicator of early lytic replication events, as well as the levels of Z, R, and tubulin (Figure 2A). Quantification of the BMRF1 protein levels relative to tubulin levels indicated that the loss of mTORC1 activity enhanced lytic replication in this cell line (Figure 2B, dark bars). Treatment of other EBV-positive epithelial cells lines (AGS-BX1 and D98/HR1) yielded similar results (Figure 2C). The rapamycin dose that had the most significant effect upon lytic replication in these cells, without impairing cell growth (5 nM) was very effective at inhibiting mTOR activity, as evidenced by the ability of this dose to inhibit the phosphorylation of the mTOR target p70S6K in these cells (Figure 2E, lane 2).As the doses used were relatively low doses of rapamycin, we tested to see if a higher dose of rapamycin would have a different effect upon lytic replication in EBV-positive epithelial cell lines, in an effort to inhibit lytic replication. We treated AGS-BDneo cells with 100 nM rapamycin for 24 hr prior to induction of lytic replication and performed Western blot analysis to examine the levels of BMRF1. We found that the higher dose of 100 nM rapamycin was still unable to inhibit lytic replication, relative to untreated (Figure 3A, lane 5).

EBV biology is cell-type specific, as the virus lytically replicates in epithelial cells to produce abundant progeny viral particles, but enters a latent state in B cells, immortalizing those cells to remain a part of those cells indefinitely [1]. To examine the effects of mTOR inhibition on EBV lytic replication in EBV-positive B cells, we treated the latently infected B cell line Raji with a dosage series of rapamycin for 24 hr then disrupted latency in these cells. Western blot analysis was performed for BMRF1, and the resulting BMRF1 levels quantified and standardized relative to tubulin levels (Figure 2A, B). In contrast to the epithelial cells, rapamycin treatment inhibited lytic replication in B cells, in a dose-dependent manner. Additional EBV-positive B cell lines were examined, including P3HR1, Daudi, and EBfaV-GFP (derived from B958), and all showed results similar to Raji, i.e. that rapamycin treatment was effective in inhibiting early lytic replication (Figure 2C). The rapamycin dose most effective in decreasing lytic replication in B cells, without impairing cell growth (5 nM), was also very effective in inhibiting the phosphorylation of p70S6K by mTOR in Raji cells (Figure 2E, lane 2).As for AGS-BDneo, we examined whether a higher dose of rapamycin would be more effective than 5 nM rapamycin at inhibiting early lytic replication in Raji cells. Figure 3B shows that 5 nM was very effective at inhibiting BMRF1 expression (Figure 3B, lane 4), and a higher dose (100 nM) was not more effective (Figure 3B, lane 5).

**Figure 4:**
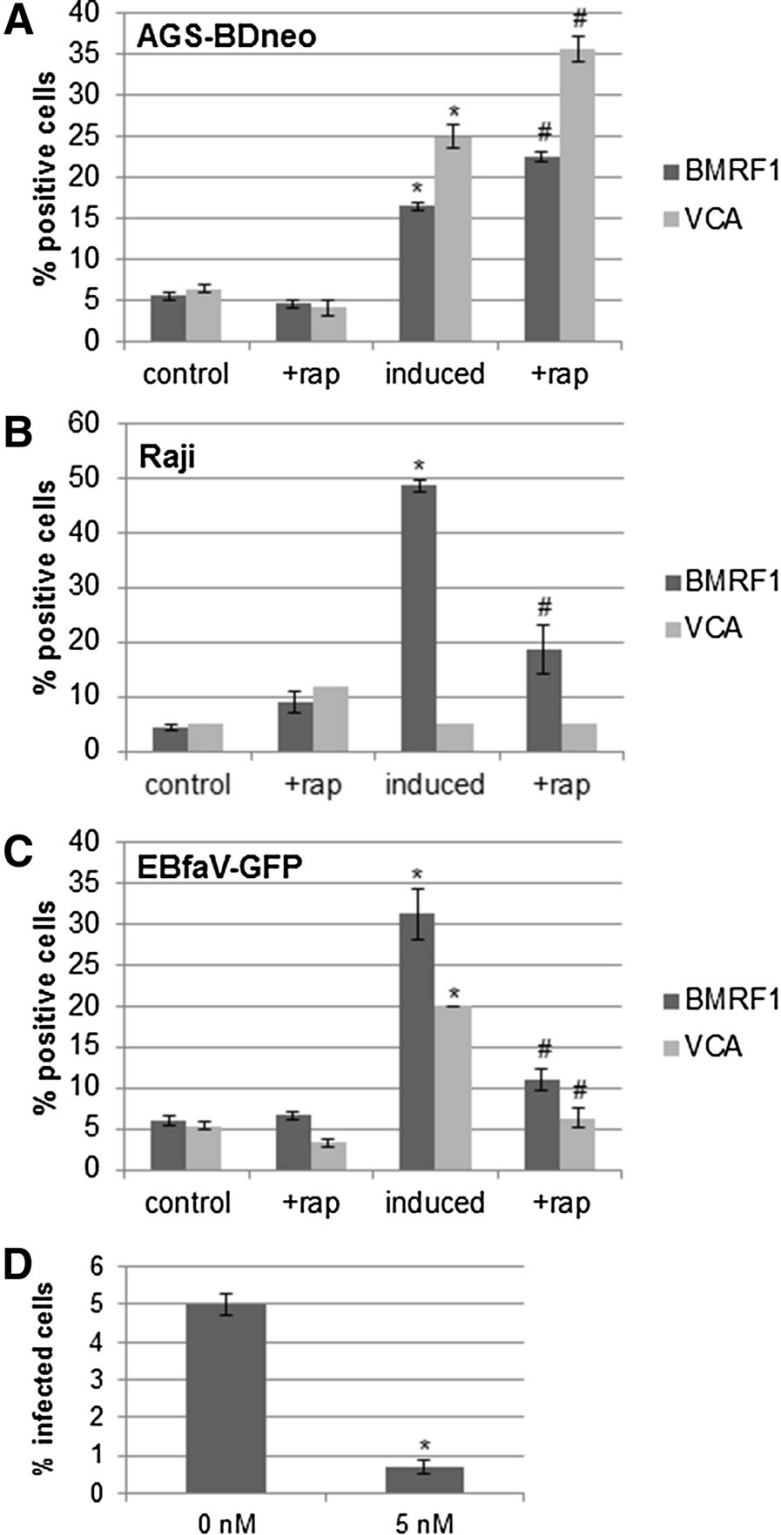
**Inhibition of mTOR alters late lytic replication in EBV-positive cells.** AGS-BDneo **(A)**, Raji **(B)**, and EBfaV-GFP **(C)** cells were left untreated or treated with 5 nM rapamycin for 24 hr prior to induction of lytic replication, as indicated. 48 hr post-infection cells were immunostained for BMRF1 or VCA, and the staining was analyzed by flow cytometry. The percent positive cells is presented. **D**. EBfaV-GFP cells were left untreated or treated with 5 nM rapamycin for 24 hr prior to induction of lytic replication, as indicated. 2 days post-induction the media from each condition was filtered and placed on Raji cells for 2 days. The number of resulting infected Raji cells was quantified, as judged by GFP, and the percent of infection presented. * = P < 0.05 relative to uninduced, untreated control cells; # = P < 0.05 relative to induced, untreated cells.

As BMRF1 expression represents early gene expression, we next examined how mTOR inhibition via rapamycin altered late gene expression. Therefore we treated the EBV-positive cell representatives AGS-BDneo and Raji with 5 nM rapamycin for 24 hr, induced the cells into lytic replication, and performed flow cytometry to assess early (BMRF1) and late (VCA) gene expression (the VCA antibody used did not recognize VCA on Western blots, thus flow cytometry was used) (Figure 4). For AGS-BDneo cells we found that, similar to the results of Figure 2, treatment with 5 nM rapamycin caused BMRF1, and VCA levels, to significantly increase in rapamycin-treated induced cells, relative to untreated induced cells (Figure 4A). For Raji cells we were not able to detect VCA (due to the lack of VCA gene expression in these cells) (Figure 4B), thus we treated the latently infected B cell line EBfaV-GFP with rapamycin and induced these cells into lytic replication, and found that rapamycin caused BMRF1, and VCA levels to significantly decrease in rapamycin-treated induced cells, in comparison to untreated induced cells (Figure 4C). To examine the end result of lytic replication, the formation of infectious viral particles, after treatment with rapamycin, we treated EBfaV-GFP cells with 0 or 5 nM rapamycin, induced the cells into lytic replication, and two days later removed the media, filtered out the cells with a 0.2 μm filter, and placed the filtered media on Raji cells for two days. The EBV virus within EBfaV-GFP harbors the GFP gene thus cells infected with this virus are GFP-positive [26]. We subsequently counted the number of resulting GFP-positive Raji cells, and found that the rapamycin treatment significantly lowered the number of viral particles being produced by EBfaV-GFP cells, as judged by the number of Raji cells infected (Figure 4D).

### Treatment of EBV-positive cell lines with rapamycin does not impact cell viability

**Figure 5:**
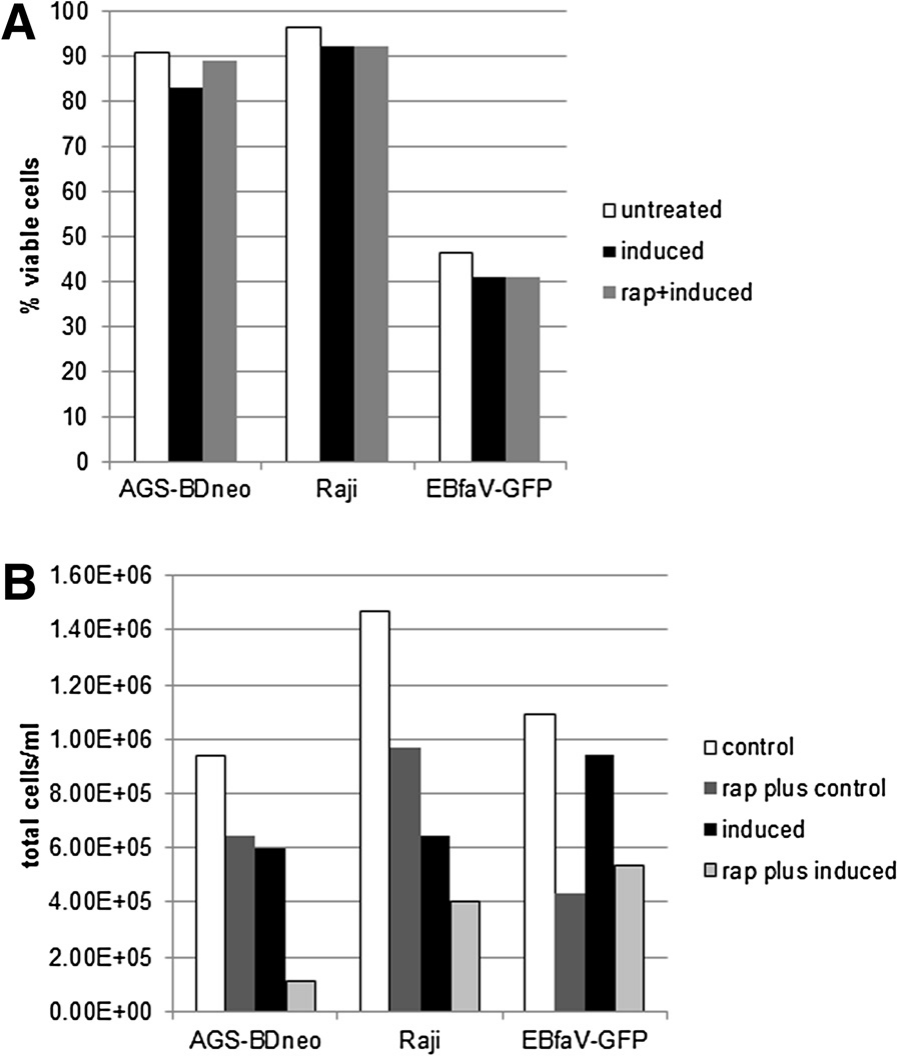
**Rapamycin treatment does not alter EBV-positive cell viability, but does decrease cell proliferation.** Raji, EBfaV-GFP, and AGS-BDneo cells were left untreated or treated with 5 nM rapamycin for 24 hr prior to induction of lytic replication, as indicated. 24 hr later the viability of cells and cell density were determined by flow cytometry. The percentage of viable cells is presented in **A**, the cell density (total cells/ml) is presented in **B**.

To ensure that rapamycin treatment did not impact the viability of the cells used in this study, we treated Raji, EBfaV-GFP, and AGS-BDneo cells with 0 or 5 nM rapamycin for 24 hr, and induced the cells into lytic replication. Viable versus dead cells were determined by flow cytometry (Figure 5A). The results indicate that rapamycin treatment did not negatively impact cell viability, thus eliminating the possibility that a loss of viability of the cells could account for the alteration of lytic replication seen above. We did find, however, that 5 nM rapamycin decreased the cell proliferation of AGS-BDneo, Raji, and EBfaV-GFP cells (Figure 5B), as would be expected [17-19].

### Short-term effects of mTOR inhibition on EBV lytic replication

**Figure 6:**
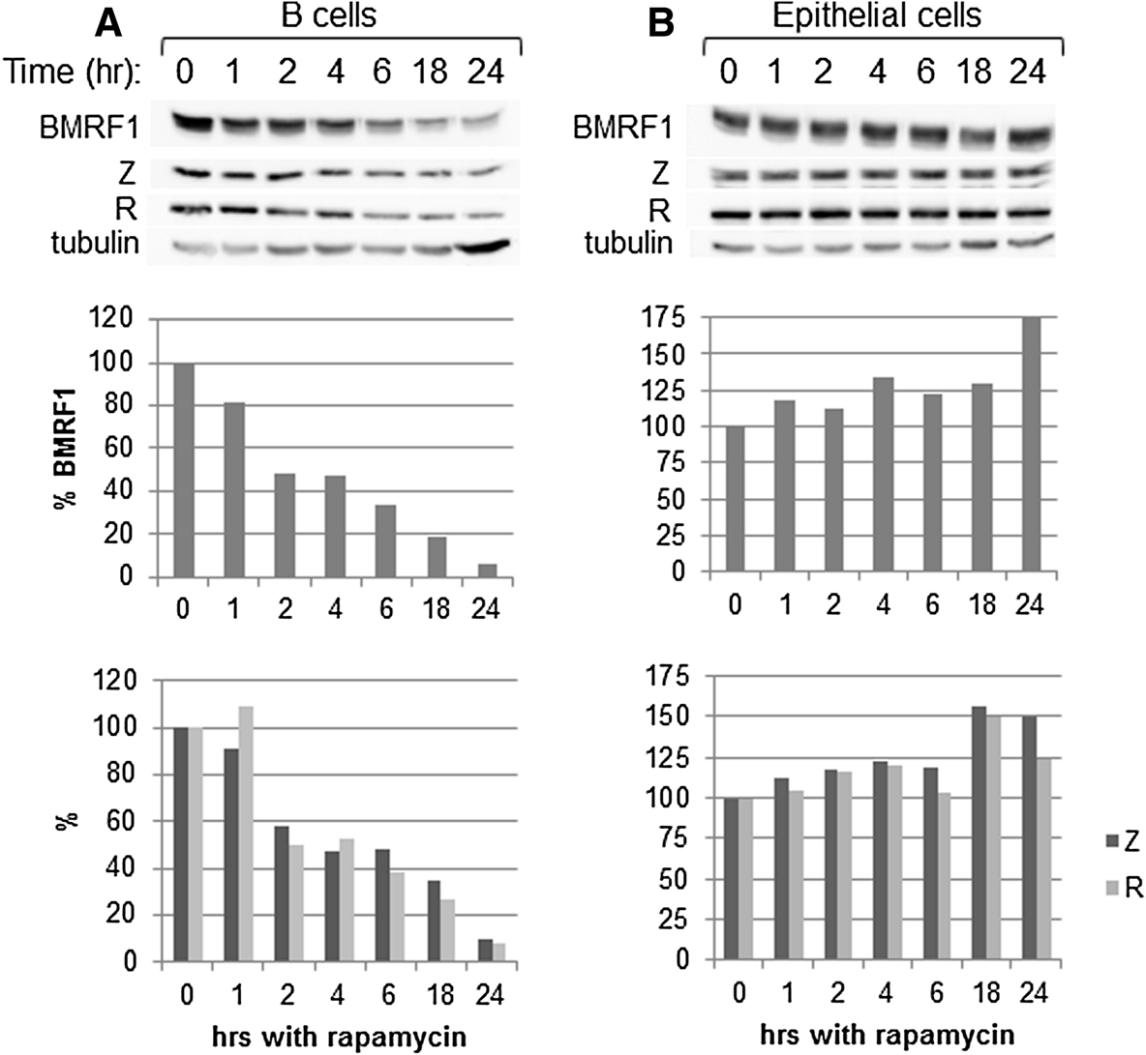
**Shorter exposure times to rapamycin are not more effective at altering BMRF1 production in either B or epithelial cells.** Raji **(A)** and D98/HR1 **(B)** cells were treated with 5 nM rapamycin for the times indicated prior to induction. The rapamycin-containing media was washed off the cells at time 0 (the time of induction). Two days post-induction proteins were harvested and Western blot analyses were performed with anti-BMRF1, anti-Z, anti-R, and anti-tubulin antibodies. The resulting bands were quantified and standardized to tubulin levels. Resulting BMRF1 protein levels (middle panels), and Z and R levels (lower panels) are presented, relative to 0 hr.

Previous studies have demonstrated that treatment of cells with low levels of rapamycin inhibits mTORC1 activity for short periods of time, then a negative feedback loop is initiated and the inhibitory effect of rapamycin is supposedly lost [27,28]. To investigate whether our dose (5 nM) of rapamycin had a different short-term effect on our cell lines, we incubated Raji cells (as a B cell representative) and D98/HR1 cells (as an epithelial cell representative) with 5 nM rapamycin for 24, 18, 6, 4, 2, 1, or 0 hr. At time 0, the cells were washed to remove the rapamycin treatment. Also at time 0 the cells were induced into lytic replication. Western blot analysis was performed to examine BMRF1, Z, R, and tubulin levels; BMRF1, Z, and R levels were quantified and standardized to tubulin levels (Figure 6). B cells that had the rapamycin washed off at time 0 (Figure 6A) had a time-dependent decrease in lytic replication, with the inhibitory effect elicited after 4 hr of treatment, continuing to decrease in a time-dependent manner until 18–24 of rapamycin treatment. Similar trends were noted with Daudi and P3HR1 cells (data not shown). Conversely, epithelial cells exhibited a time-dependent increase in lytic replication, with the strongest effect at 24 hr of rapamycin treatment (Figure 6B). Therefore, shorter exposure times did not show a different course of action of rapamycin in regard to lytic replication in either of these cell types.

For all cell lines tested, treatment with rapamycin 18–24 hr prior to induction yielded the most significant results. Rapamycin treatment at the time of induction (0 hr) yielded a milder change, and treatment 24 hr post-induction yielded little to no change in BMRF1 levels (data not shown).

### Z and R transcript and protein levels are altered by rapamycin treatment in a cell-type dependent manner

**Figure 7:**
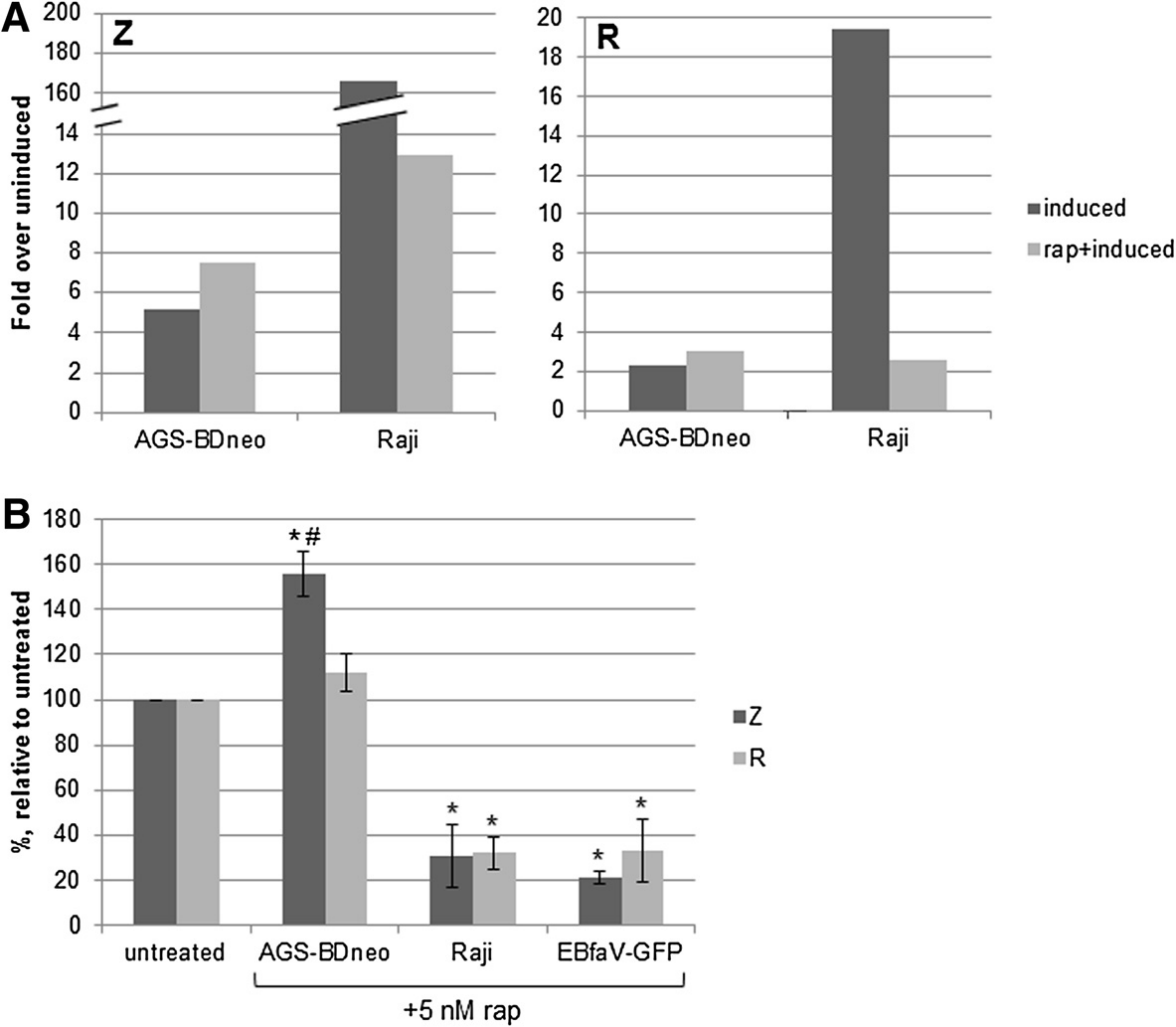
**Z and R transcript and protein levels are altered by rapamycin treatment.** Raji and AGS-BDneo cells were treated with 5 nM rapamycin for 24 hr prior to induction of lytic replication. **A**. Total RNA was isolated and qRT-PCR was performed with GAPDH (control)-, Z-, or R-specific primers, in triplicate. The mean fold expression over uninduced cells, standardized to GAPDH levels, is presented. **B**. Z and R protein levels from Western blots (in triplicate) were quantified and standardized to tubulin levels. The resulting Z and R protein levels from AGS-BDneo, Raji, and EBfaV-GFP cells are presented, relative to induced cells (with no rapamycin treatment). * = P < 0.05 relative to induced cells (with no rapamycin treatment); # = P < 0.05 relative to R levels within same cell type.

Our initial work in *Drosophila* suggested that loss of *Tor* activity in *Drosophila* eye cells led to altered Z and R activity (greater Z activity and reduced R activity), with the most significant impact on Z activity (judging by the severity of the phenotypes in Figure 1). Thus we next sought to examine whether inhibition of mTOR in EBV-positive cells could alter expression levels of Z or R. To this end we treated the EBV-positive cell lines Raji and AGS-BDneo with 5 nM rapamycin, induced cells into lytic replication, and subsequently harvested RNA from the cells. Quantitative RT-PCR was performed (Figure 7A). We found that while rapamycin treatment had no/little effect upon the level of GAPDH (control) transcripts, rapamycin treatment did affect transcript levels of both Z and R, in a cell-type dependent manner (Figure 7A). In the Raji B cell line, induction alone caused an average 166-fold increase in Z transcripts over uninduced cells; this was decreased to 13 fold when cells were pre-treated with 5 nM rapamycin. Similarly, induction caused a nearly 20 fold increase in R transcripts in Raji cells; this was decreased to approximately 2 fold after rapamycin treatment. Conversely, in the AGS-BDneo epithelial cell line, rapamycin treatment increased both Z and R transcript levels, from an average of 5 fold to about 8 fold for Z, and an average of 2 to 3 fold for R, in relation to uninduced cells. It is worth noting that while it appears that Z and R transcripts are much more abundant in Raji cells than epithelial cells (Figure 7A), there actually was a higher baseline of Z and R transcript levels present in AGS-BDneo cells, including the uninduced cells, than in Raji cells. By qRT-PCR we found that while the GAPDH control transcript levels varied between AGS-BDneo and Raji by a ΔCT of 0.6 (representing a 1.5 fold increase of GAPDH transcript in the AGS-BDneo cells), the Z transcript levels differed by a ΔCT of 7.8 between uninduced AGS-BDneo and Raji cells (representing an over 200-fold increase of baseline Z transcripts in the AGS-BDneo cells). The same trend was found for R transcript levels in AGS-BDneo versus Raji cells.The Z and R transcript levels correlated with subsequent protein levels, such that rapamycin treatment significantly reduced Z and R protein levels in Raji cells (and EBfaV-GFP cells) (Figure 7B). Rapamycin treatment of AGS-BDneo cells increased Z and R protein levels; Z demonstrated a significant increase, while R levels were typically increased only moderately (Figure 7B). For rapamycin-treated AGS-BDneo cells the levels of Z were significantly higher than those of R, suggesting that the increase in lytic replication in these cells may be due more to rapamycin-mediated effects upon Z rather than R. Rapamycin-based changes in Z and R expression levels were also demonstrated in Figure 2A (shown quantified in 2D) as well as in Figure 6A and B.

## Discussion

We discovered that modulation of mTOR activity alters EBV lytic replication, beginning with a genetic screen in *Drosophila* that demonstrated that loss of *Tor* enhanced Z activity, and suppressed R activity, in *Drosophila* tissues (Figure 1). It logically followed that inhibition of mTOR in human cells would alter the lytic replication of EBV, as lytic replication is dependent upon both Z and R activities.We found that the rapamycin-mediated inhibition of mTOR had varying effects upon lytic replication amongst different EBV-positive cell lines. Inhibition of mTOR inhibited lytic replication in B cell lines, while in contrast enhanced lytic replication in epithelial cell lines (Figures 2 and 4). The effects upon lytic replication were not time-dependent, as would be found if the action of rapamycin was negated by a negative feedback loop, as short-term doses of rapamycin yielded the same results as longer-term doses [i.e. in B cells rapamycin inhibited lytic replication at all time points, and in epithelial cells rapamycin enhanced lytic replication at all time points (Figure 6)]. Furthermore, in an attempt to have epithelial cells mimic the B cell response to rapamycin, we treated epithelial cells with higher doses of rapamycin, yet lytic replication in these cells was still enhanced by the higher doses (Figure 3).

As the immediate-early proteins Z and R are responsible for initiating lytic replication, and lytic replication was altered upon rapamycin treatment, it follows that the inhibition of mTOR likely altered these proteins’ levels or functions. In the case of KSHV, Nichols et al. found that treatment of KSHV-positive cells with 12 nM rapamycin inhibited the KSHV immediate-early protein RTA from being translated [20]. This logically led to a loss of lytic replication in these cells [20]. However for other herpesviruses such as human cytomegalovirus, treatment with 20 nM rapamycin or 250 nM Torin1 had little effect upon immediate-early protein levels, yet caused a decrease in the number of viral particles produced via lytic replication [21]. Therefore, it does not appear that inhibition of mTORC1 simply leads to an inhibition of viral protein translation for all viruses. In our study we found that rapamycin treatment actually altered the transcript levels of Z and R, such that in B cells Z and R transcript levels were decreased when lytic replication was induced in the presence of rapamycin, while in epithelial cells Z and R transcript levels were increased when lytic replication was induced in the presence of rapamycin (Figure 7A). The resulting Z and R protein levels correlated with the transcript levels (Figure 7B), and with the amount of Z protein binding to Z-response elements (ZREs; data not shown); this correlates with the levels of lytic replication that ensued in each cell type.

**Figure 8:**
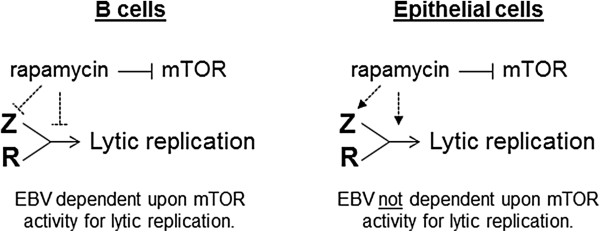
Model of rapamycin action upon EBV lytic replication.

Thus it appears that mTORC1 activity plays a role in lytic replication at least in part by regulating the expression of the Z and R genes. mTORC1 activity has been shown to have a significant role in regulating the activity of a variety of transcription factors (reviewed in [29]), including at least one transcription factor (YY1) that is known to regulate Z and R promoter activation [30,31]. That inhibition of mTORC1 yields opposing effects in the transcription of Z and R in B versus epithelial cell lines may have to do with different transcription factor activities in these different cell lines, and how such transcription factors are affected by loss of mTOR activity.A working model based on our study is presented in Figure 8. Overall, we have found that B cells require active mTORC1 for efficient lytic replication. In contrast, epithelial cells do not require active mTORC1 to engage in productive lytic replication.

## Conclusions

mTOR function is central to many different cellular processes, and viruses have evolved to manipulate the mTOR pathways to aid their replication [32,33]. Our investigations have provided evidence that EBV replication is dependent upon the mTOR pathway, at least in B cells, and that inhibiting this pathway in B cells greatly limits EBV lytic replication. However direct inhibition of mTOR in epithelial cells has the opposite effect, and enhances EBV lytic replication. These results are important to take into account when considering the use of mTOR inhibitors as anti-viral or anti-cancer therapeutic agents.

## Materials and methods

### SEMs

Flies were sputter coated with gold in a Pelco model 3 sputter coater 91000. Images were taken on a Hitachi S-4800 SEM microscope.

### Cell lines

D98/HR1, a latently infected, EBV-positive epithelial cell line created by the fusion of HeLa cells with EBV-positive P3HR1 cells were maintained in Ham’s F12 medium supplemented with 10% fetal bovine serum along with penicillin, streptomycin, and fungicide. AGS-BDneo and AGS-BX1 are EBV-positive gastric carcinoma cell lines (gifts of L. Hutt-Fletcher) and were maintained in Ham’s F12 supplemented with 10% fetal bovine serum along with penicillin, streptomycin, fungicide, and 500 μg/ml G418. Raji, Daudi, and P3HR1 (ATCC) are latently infected, EBV-positive B cell lines derived from Burkitt’s lymphoma. EBfaV-GFP is a derivative of the B958 cell line, in which the viral LMP2 gene was replaced by the EGFP gene driven by the CMV immediate-early promoter [26]. All B cell lines were maintained in RPMI supplemented with 10% fetal bovine serum along with penicillin, streptomycin, and fungicide.

### Cell treatments

Cells were treated with rapamycin (in DMSO, Sigma-Aldrich) for the times indicated. Cells not treated with either compound were treated with the vehicle, DMSO (Sigma-Aldrich). If cells were washed prior to induction, cells had their media removed, were washed one time with 1xPBS, then regular media added back. If unwashed, then the rapamycin or DMSO remained present in the media until cells were harvested.

### Disruption of viral latency

B cells were treated with 20 ng/ml TPA and 3 mM sodium butyrate, and epithelial cells were treated with 5 ng/ml TPA and 0.75 mM sodium butyrate (higher doses of these inducing chemicals caused significant cell death of our AGS cell lines) for 24–48 hr as indicated (both from Sigma-Aldrich).

### Protein preparation

Cells were collected, washed twice with 1xPBS, resuspended in ELB lysis buffer (0.25 M NaCl, 0.1% NP40, 50 mM HEPES pH 7, 5 mM EDTA and protease inhibitors), and sonicated. The lysed cells were centrifuged and the supernatants quantified for protein concentration prior to SDS-PAGE.

### Western blotting

Equal amounts of protein (20–40 μg) were loaded onto 10% SDS-PAGE gels and electrophoresed. Proteins were transferred to Immobilon (Millipore) membranes.

### Immunoblotting

Blots were blocked with blocking buffer (5% nonfat dried milk, with 0.1% Tween-20, 1xPBS) for 2 hr to overnight. Primary antibodies: anti-BMRF1 (Capricorn), anti-tubulin (Developmental Hybridoma Bank), anti-R (Argene) and anti-Z (Argene or Santa Cruz) were diluted 1:200 in blocking buffer; anti-phospho-p70S6K and anti-total p70S6K (Cell Signaling) were diluted 1:1000 in blocking buffer; all were incubated with the blots 1 hr to overnight, washed with 0.1% Tween-20 in 1xPBS 3 times for 10 min each, and incubated in secondary antibody (goat-anti-mouse HRP or goat-anti-rabbit-HRP, Jackson Immunoresearch) diluted 1:10,000 in blocking buffer, for 1 hr. Blots were washed as before and visualized with Super Signal (Pierce). Images and quantification were performed with a BioRad gel documentation apparatus.

### Viability and cell density testing

Cells were treated with rapamycin for 24 hr then induced. Cells were collected and diluted in Guava ViaCount solution. After a five minute incubation, cell viability and cell density (total cells/ml) were determined by flow cytometry with a Guava InCyte flow cytometer.

### Flow cytometry

Cells were treated as indicated, washed with PBS, and fixed with 60% acetone in PBS for 10 min at 4°. Cells were washed with PBS/0.5% BSA, incubated with anti-BMRF1 (Capricorn) or anti-VCA (Argene) antibodies [1:200 in Incubation mix (0.3% BSA, 5% goat serum, 0.1% Triton X in PBS)] for 1 hr at room temperature, washed with PBS/0.5% BSA, and incubated in donkey-anti-mouse-FITC (1:400 in Incubation mix) (Jackson Immunoresearch) for 1 hr at room temperature. Cells were washed with PBS/0.5% BSA, resuspended in PBS, and analyzed with a Guava EasyCyte flow cytometer (Millipore).

### qRT-PCR

Total RNA from treated cells was isolated with the RNeasy mini kit (QIAGEN). qRT-PCR reactions (20 μL) were performed with the Power SYBR Green RNA-to-C_T_™ 1-Step kit (Applied Biosystems), 10 ng of total RNA, and 20 pmol of each primer, as per the manufacturer’s directions. Primers used for Z were: 5’-ACTGCTGCAGCACTACCGTGAGGTG-3’ (forward) and 5’-GAAATTTAAGAGATCCTCGTCTAA-3’ (reverse) (creating a 150 bp product). Primers for R were: 5’-ATGAGGCCTAAAAAGGATGGCTT-3’ (forward) and 5’-TGAGGACGTTGCAGTAGTCAG-3’ (reverse) (creating a 100 bp product). Reactions were performed and analyzed with an Applied Biosystems Step One Plus real time PCR machine.

### Quantification and statistics

Western blots were quantified with ChemiDoc XRS software (BioRad) and flow cytometry data was quantified with Guava EasyCyte analysis software (Millipore). Experiments were done in triplicate, and the student T-test (two-tailed) used to evaluate significance. A P value <0.05 was considered significant.

## Competing interests

The authors declare that they have no competing interests.

## Authors’ contributions

BTL carried out the quantitative RT-PCR. BDS carried out immunoassays. ALA conceived of the study, participated in its design and coordination, performed the SEMs, immunoassays, and flow cytometry, and wrote the manuscript. All authors read and approved the final manuscript.
